# Breastfeeding in a Polluted World: Perspective on the Properties of Breast Milk and the Need for Protection

**DOI:** 10.3390/jcm14228034

**Published:** 2025-11-13

**Authors:** Maria Elisabeth Street, Anna-Mariia Shulhai, Vassilios Fanos, Anna Maria Papini, Davide Ponzi, Antonio Ragusa, Dolores Rollo, Paola Palanza

**Affiliations:** 1 Department of Medicine and Surgery, University of Parma, 43121 Parma, Italy; annashulhai@gmail.com (A.-M.S.); davide.ponzi@unipr.it (D.P.); dolores.rollo@unipr.it (D.R.); paola.palanza@unipr.it (P.P.); 2 Unit of Paediatrics, University Hospital of Parma, 43126 Parma, Italy; 3 Section of Neonatal Intensive Care Unit, Department of Paediatrics, Puericulture Institute and Neonatal Section, Azienda Mista, University of Cagliari, 09124 Cagliari, Italy; vafanos@tiscali.it; 4 Interdepartmental Research Unit of Peptide and Protein Chemistry and Biology (Peptlab), Centre of Competences in Molecular Diagnostics and Life Sciences (MoD&LS), University of Florence, 50121 Sesto Fiorentino, Italy; annamaria.papini@unifi.it; 5 Department of Obstetrics and Gynecology, Hospital of Sassuolo, 41049 Modena, Italy; antonio.ragusa@gmail.com

**Keywords:** breast milk, human milk, human health, biomonitoring, environmental exposure, endocrine-disrupting chemicals, microplastics, nanoplastics, analytical chemistry, toxicology

## Abstract

Breast milk (BM) is a unique biological fluid that represents the optimal nutritional source for infants, uniquely adapted through millions of years of evolution. BM is not only a nutritional fluid but a dynamic biological system, evolved to provide optimal growth, immune protection, and neurodevelopmental support. Its unique composition—including macronutrients, micronutrients, bioactive molecules, and stem cells—makes it essential in early life. Breastfeeding further promotes psychological well-being, secure attachment, and maternal–infant bonding. Yet, in recent decades, concern has grown over environmental contaminants in BM, including endocrine-disrupting chemicals (EDCs) and micro/nanoplastics. These pollutants have the potential to disrupt endocrine signaling, neurodevelopment, metabolic programming, and immune development, thereby undermining the natural advantages of breastfeeding. Therefore, a better understanding of the unique features of BM, while investigating the effects of these contaminants, is important for safeguarding maternal and infant health. This perspective article highlights the current knowledge on BM and indicates the need for further research. It also emphasizes the need for appropriate public health measures aimed at reducing exposure to pollutants and lowering associated risks, as well as preventive strategies to protect breast milk and breastfeeding in such a changing environment, as it is uniquely designed to promote the health of children.

## 1. Introduction

### Evolutionary Evidence and Psychological Functions of Breast Milk and Breastfeeding

Mammary glands give their taxonomic name to mammals for their relevance. They probably first developed during the Jurassic age from glandular skin structures and worked as moisturizers for egg supportive structures or as a source of nutrition for altricial offspring [1]. Since milk composition has remained relatively constant over time, regardless of food availability, lactation has buffered offspring’s nutritional risk, allowing mammals to adapt to different ecologies. With evolution, lactation has prolonged the period of post-natal maternal care and of lifespan, besides leading to complex sociality and larger brains with respect to body size, although with some variability depending on species [2].

In humans, breastfeeding is a multifaceted experience that, in addition to nutritional and physical benefits, has important psychological effects on infants and their mothers. Abundant empirical evidence has proved that breastfeeding has positive effects on the brain, cognitive and socioemotional development, and favors mothers’ well-being [3,4].

An embodied and relational commitment between mother and child is already present prenatally. This is an intersubjective exchange that at birth is enriched by direct body-to-body contact and by gazing [5]. Intersubjectivity is an innate capacity that enables reciprocal and synchronous physical and mental exchanges between the infant and its caregiver: breastfeeding primarily strengthens the intersubjective capacity of both the mother and child [6].

In humans, the interest in breastfeeding’s psychological effects, particularly its connection to attachment, has increased recently [4,7]. Exclusive breastfeeding has been associated with a more secure attachment between the infant and mother, but these associations are also indirect, through maternal sensitivity and emotional availability. While touching calms the infant, physical closeness and direct skin contact facilitates greater awareness of the infant’s nonverbal cues, in the mother, fostering an intuitive understanding of the babies’ needs [8,9]. Breastfeeding contributes to the development of secure attachment through consistent and responsive interactions between the caregiver and the infant [10]. These interactions foster a sense of intimacy and connection in the dyad, helping the infant build a strong emotional bond [11]. Researchers have attributed this association to both physiological and psychological explanations. Breastfeeding increases oxytocin and prolactin production [12], which lower maternal stress [13], reduce postpartum depressive symptoms, and favor a higher self-esteem [14] and expression of positive emotions [15].

Finally, several findings suggest that the somatosensory stimuli during breastfeeding activate parasympathetic activity in the mothers [16] increasing heart rate variability in the infants [13]. This was proven in one study [17].

Breast milk (BM) is a unique and extraordinary nourishment and has many properties that have yet to be discovered. BM shapes the rest of life by setting in motion the body’s metabolic processes and is able to influence and govern all the physiological processes, including growth and development, microanatomy and functions, physiology and metabolism, colonization and maturation of the microbiome, immunological orientation and consolidation, and brain development and organization [17,18].

Recent studies have proved that breast milk is also an excellent substrate to study environmental exposure, and it contains pollutants accumulated in the mother’s body from the environment. Exposure varies in relationship to geographical regions, seasons, lifestyle and nutritional habits [19,20].

Therefore, this perspective article summarizes current knowledge on essential functions of BM and the challenges related to environmental contamination, including endocrine-disrupting chemicals and micro- and nanoplastics.

This perspective article is based on a non-systematic synthesis of recent literature, conducted through PubMed and Scopus searches of studies published up to July 2025, and on expert experience from researchers working in the field of breast milk and environmental contaminant research.

## 2. The Unique Features of Breast Milk

BM represents one of the most individualized biological substances in nature as it changes over time as needed (colostrum, transitional milk, mature milk), during the day (in the morning milk is more energy-rich than in the evening), and finally during a single feeding (at the end of the meal, the fats that saturate the newborn’s satiety centers increase) [18,21].

Beyond providing essential macronutrients and micronutrients, BM contains specific bioactive compounds that program the development of cognitive capabilities and their neurobiological determinants [18,21]. These include long-chain polyunsaturated fatty acids (LC-PUFAs), choline [22], neurotrophic factors [23], sialic acid and oligosaccharides that play an important role for synaptogenesis and neuronal signaling [24], hormones and cytokines that modulate neuroimmune interactions and brain development.

Along with already known data, Microbiomics, Metabolomics, and Multipotent Stem Cells, referred to as the 3Ms of BM, should be emphasized.

### 2.1. Microbiome

In the past, BM was thought to be free of bacterial colonization, and this was considered one of the advantages of breastfeeding. Today, we know that breast milk contains bacteria (microbiota), specifically at least 50 genera and 200 species which come from sources both inside and outside the breasts [25].

Similarly to the placenta that has a microbiota very similar to that of the mother’s tongue and throat, the microbiota of BM is very close to that of the mother’s intestine [26,27]. If the mother has a dysbiosis she might transfer bacteria that are not completely beneficial to her newborn [28]. Interestingly, the microbiota found in BM of mothers who have undergone an emergency cesarean section is more similar to that of women who have had a vaginal delivery, while it differs from the microbiota of mothers who have given birth via planned cesarean section, likely due to stress. These changes are thought to play currently an important role on the overall health of infants [29].

### 2.2. Metabolomics

BM has been considered miraculous since ancient times, and it is not surprising that comparing the metabolomic data of breast milk at 1 and 4 weeks after birth, numerous substances vary in quantity during the first month of life, for example, oleic, linoleic, and palmitoleic acids increase, while cholesterol decreases [30]. Moreover, the milk of mothers who have given birth to very low gestational age infants differs from that of mothers of late preterm infants. The former BM contains lower levels of α1,3-linked fucosyl residues, choline, 3′sialyllactoses, glucosyl moieties, and myo-inositol in its metabolome when compared to the BM of late preterm mothers [30].

In addition, the milk of mothers with major obstetric syndromes, or obesity, has been studied showing important differences in aromatic amino acid and derivatives, acyl-carnitines, lipids and oligosaccharide contents [31].

Recent studies have made significant contributions in identifying “secretory” and “non-secretory” women based on the presence or absence of fucosylated human milk oligosaccharides, which influence selective growth of Bifidobacteria and Bacteroides in the infant gut that have been shown to play a protective role in HIV transmission [31].

### 2.3. Multipotent Stem Cells

BM has been shown to contain stem cells that can cross both the intestinal and the blood-brain barriers, migrate into the brain and other organs and systems, including the thymus, pancreas, and liver. Once they reach their destination within the newborn’s tissues, they differentiate, assimilating and integrating with the newborn’s tissues. In the brain, stem cells can differentiate into neurons, astrocytes, and oligodendrocytes [32].

## 3. Breast Milk Reflects the Environment We Live in

### 3.1. Endocrine-Disrupting Chemicals in BM and Their Effects

Endocrine-disrupting chemicals (EDCs) are man-made chemicals that interfere with hormonal regulation by mimicking, blocking, or altering hormone synthesis, secretion, transport, signaling, and action [19,33,34]. Some of these chemicals have a lipophilic nature and can bioaccumulate in adipose tissue in mothers, being then transferred first to the fetus through the placenta and then to newborns and infants via lactation [33,34]. Both fetal life and infancy, the first 1000 days of life, represent critical windows of developmental vulnerability. The most well-known and common EDCs include phthalates, bisphenols, per- and polyfluoroalkyl substances (PFAS), flame retardants, and persistent organic pollutants (POPs). These can be found in everyday life products, such as personal care items, food and beverages, packaging, cleaning supplies, agriculture chemicals, products used for covering, solvents, etc. [33].

The endocrine system is particularly sensitive to disruptions during early life, and EDC exposure can affect key endocrine axes, including thyroid function, puberty regulation, reproductive programming, bone health, metabolic homeostasis, and adipogenesis [19,33].

Recent studies have evidenced that high exposure to some pollutants could reduce BM benefits, as for the case of PFAS [35]. These are highly persistent chemicals that accumulate in the human body, earning the name of “forever chemicals”, and have been linked to adverse health effects including immune dysfunction, thyroid disruption, and increased cancer risk [33]. Human studies have shown that high PFAS exposure can be associated with growth impairment, however, interestingly, the benefits of exclusive breastfeeding during the first three months of life appear to outweigh the potential adverse effects associated with elevated plasma PFAS levels observed during the first two years of life [35].

Previous studies found Bisphenol A (BPA) and polycyclic aromatic hydrocarbons (PAHs) in formulas and BM, with PAH levels in some areas of Southern Italy being associated with petrogenic and pyrolytic environmental sources, such as waste incineration, indicating maternal exposure through the environment during breastfeeding [36]. The presence of metals like aluminum, arsenic, and cadmium in milk also can be attributed to environmental contamination of soil or grasslands. PFAS levels have been noted to be slightly higher in BM compared to infant formula. The presence of phthalate monoesters in formula could also stem from cow’s milk, which can incorporate these pollutants [36].

Epidemiological and experimental studies have shown that phthalates, phenols, bisphenols, PFAS, perchlorate, polychlorinated biphenyls (PCBs), dioxins, thiocyanate, may modify thyroid function [37] with subsequent effects on metabolism, growth, and brain development calling for awareness and the need to reduce exposure to protect BM.

Animal studies have shown that early-life exposure to compounds as dioxins, BPA, and phthalates can impair bone mineralization, reduce bone density, and alter growth plate development [38,39,40]. Epidemiological data in humans on long-term skeletal effects of EDC exposure are yet missing [39].

Some EDCs are recognized as obesogens as they have adipogenic effects, favor insulin resistance, cause metabolic and gut microbiome changes, alter energy balance, and induce epigenetic changes [41,42]. The impact of EDC exposure via BM on obesity and metabolic changes is supported by a few prospective studies [41]. Nevertheless, exclusive breastfeeding during the first 6 months of life leads to significant DNA variants, which may play a protective role in preventing overweight and obesity in children [43]. Although there are some studies on single chemical exposure, research on the impacts of chemical mixtures, which reflect real-life exposure, remains limited.

### 3.2. EDCs in BM and Neurodevelopment

Due to evolutionary trade-offs related to bipedalism, human newborns are born with underdeveloped brains that are only about 25 percent of their adult size, less than in any other mammal [44]. Consequently, 75% of human brain growth occurs postnatally and mostly during the first year of life, which represents a highly critical period of development characterized by elevated neural plasticity and sensitivity to environmental inputs, including nutritional, chemical and social factors. These influences can significantly impact developmental trajectories and future health outcomes [45].

Despite the heterogeneity of current studies, there is robust evidence of associations between breastfeeding (especially exclusive and longer duration) and higher IQ, better academic performance, and improved brain structure [46]. Children who were not breastfed presented a lower verbal intelligence quotient at 30 years of age compared to age-matched controls fed exclusively with BM for 6 months or more [47]. A few studies have suggested protective effects of breastfeeding against neurodevelopmental disorders such as attention deficit hyperactivity disorder (ADHD) symptoms and autism spectrum disorder (ASD) traits although findings are mixed and confounded by genetic and socio-economic factors [48,49].

Newborns and infants in the first year(s) of life are particularly vulnerable to environmental pollutants, as EDCs, because the nervous system is rapidly developing, the blood barrier is still immature, and hormones play a crucial role in brain developmental trajectories [50]. Accumulating evidence indicates that early-life exposure to EDCs increases the risk of neurodevelopmental disorders, including ADHD, ASD, intellectual disabilities, and more generally is associated with subclinical deviation from neurotypical development [51,52].

The presence of single and/or mixtures of EDCs in BM has been shown to impact negatively on infants’ cognitive, language, motor, socio-emotional, and behavioral development [20]. More specifically, PCBs and dioxins in BM have been associated with decreased cognitive development and psychomotor responses in children [20]. Recent studies have linked early life PFAS exposure to reduced cognitive, motor, and language development in infancy and hyperactivity in childhood [53,54], though not all the studies specifically addressed the issue of BM exposure. Sex-specific associations have also been reported but findings are not consistent throughout the different studies. An interesting finding is that most studies concerned low-level EDC exposure, suggesting subtle but potentially significant population-wide neurodevelopmental effects.

The heterogeneity of the current literature, partially due to the great variability in methodology and procedures (i.e., chemicals, timing of sampling, neurodevelopmental endpoints, age, populations, etc.), makes it difficult to harmonize and compare findings on the impact of environmental exposure to EDCs in breastmilk on infant neurodevelopment. There is a clear need for consistent longitudinal studies that focus on BM as a critical matrix for monitoring maternal and infant EDC exposure, examining its effects on brain and behavioral development via breastmilk or formula milk. Therefore, research on protective strategies to reduce maternal EDC exposure should be strongly supported to ensure prevention and promote public health.

### 3.3. Measuring EDCs in Milk

BM as previously mentioned is recognized to date as a critical substrate for evaluating environmental exposure to contaminants, particularly EDCs.

The characteristics that make BM a valuable biomarker include its direct route of exposure for neonates and its ability to reflect the mother’s overall exposure to a wide array of chemicals [19,55]. However, there are significant criticisms and challenges related to the dosages of EDCs in milk. The complex matrix of milk (not only BM), rich in proteins and lipids, requires sophisticated analytical methods for extraction and quantification. Ultra-Performance Liquid Chromatography–Tandem Mass Spectrometry (UPLC-MS/MS) methodologies and one ICP-AES method that have been developed allow currently to quantify 85 different chemicals, most of these already accepted as EDCs, some alerted, in any matrix including the most complex biologically [36].

In general, several pitfalls can arise when analyzing exogenous chemicals in biological matrices. Traditional approaches such as liquid–liquid extraction and solid-phase extraction remain the most widely used techniques for isolating compounds from biological samples, though novel, environmentally friendly microextraction methods are under active development [56]. When employing tandem mass spectrometry, a powerful analytical tool typically coupled with liquid chromatography or gas chromatography for quantitative analyses, the issue of “matrix effects” becomes critical. This term describes the discrepancy between the mass spectrometric response of an analyte in pure solution versus complex biological media such as urine, plasma, or serum. These effects, caused by endogenous components or preservative agents, can alter chromatographic performance and ionization efficiency, leading to ion suppression or enhancement. Their magnitude depends on the ionization technique, sample preparation method, and the nature of the biological matrix [57]. Considering these challenges, it is essential that clinicians collaborate closely with chemists to develop standardized procedures tailored to specific matrices and technologies, ensuring reliable diagnostics chemicals’ diagnostics and to prevent bias in clinical studies.

In this context, controlling laboratory background contamination is also crucial, especially for ubiquitous substances such as phthalates, parabens, and bisphenols, which are present in almost all laboratory equipment, solvents, and air. Additionally, the methods used for collecting and storing samples are equally important.

The regulatory assessment of endocrine disruptors (EDs) within the European Union has been shaped by the joint European Food Safety Authority (EFSA)—European Chemicals Agency (ECHA) guidance [58]. This guidance outlines a stepwise approach requiring demonstration of endocrine activity, adverse effects, and a biologically plausible link between these through a mode of action analysis. The framework applies equally to human health and non-target organisms, with particular attention to EATS (estrogen, androgen, thyroid, and steroidogenesis pathways) modalities. It emphasizes the use of systematic evidence collection and a weight-of-evidence approach to integrate diverse data streams into regulatory decision-making.

ECHA revised the Classification, Labelling and Packaging guidance to formally include new hazard classes for EDs in November 2024 [58]. Two categories were defined: Category 1 for known or presumed EDs, where strong evidence exists for endocrine activity and adversity with a biologically plausible link, and Category 2 for suspected EDs, supported by less conclusive but still coherent evidence [58].

Therefore, an increasing number of environmental chemicals measured in formula and BM requires continuous biomonitoring and an adjustment of safety limits of exposure.

### 3.4. Micro- and Nanoplastics in Human Breast Milk

Microplastics (MPs; <5 mm) and nanoplastics (NPs; <1 µm) are increasingly recognized as emerging contaminants of concern due to their persistence, widespread distribution, and potential for human exposure. These synthetic particles, originating from the breakdown of plastic products or direct emission from industrial processes, have been detected in diverse environmental compartments and, more recently, in human biological fluids, including BM [59]. This raises critical questions on maternal and neonatal exposure during key developmental windows.

Preliminary studies have reported the presence of MPs and NPs in breast milk samples from healthy lactating women [59], suggesting that maternal exposure via diet, personal care products, and ambient air may lead to systemic uptake and potential neonatal exposure through lactation. However, current data are scarce, and the reliability and comparability of findings are constrained by significant methodological heterogeneity and the lack of standardized protocols [60].

Detection techniques such as Fourier-transform infrared and Raman spectroscopy allow for chemical identification but are limited by particle size and matrix interference. Pyrolysis–gas chromatography–mass spectrometry (Pyr-GC-MS) offers quantitative polymer profiling but is destructive and lacks morphological context. Electron microscopy provides detailed imaging but requires extensive preparation and cannot independently verify chemical composition. A harmonized, validated approach combining physical and chemical characterization is urgently needed [61].

Understanding the transfer and impact of MPs and NPs through breast milk is vital in the broader context of environmental toxicology and public health. Protecting breastfeeding as a foundational practice for infant nutrition and immunity requires a science-driven assessment of emerging contaminants. Multidisciplinary collaborations between toxicologists, environmental scientists, clinicians, and regulatory agencies will be essential to address current knowledge gaps and guide future preventive strategies.

## 4. Conclusions and Future Perspectives

Breast milk is the natural and optimal source of nutrition for infants and offers countless nutritional and immunological benefits, along with significant psychological advantages including maternal-infant bonding, which fosters secure attachment and emotional regulation during early childhood. Furthermore, BM is important for optimal neurodevelopment, behavioral, and cognitive development. The World Health Organization (WHO) correctly encourages the implementation of activities “to protect, promote and support exclusive breastfeeding for 6 months as a global public health recommendation, and to provide safe and appropriate complementary foods, with continued breastfeeding for up to 2 years of age or beyond” [62].

Given the potential risks associated with environmental pollutants, breastfeeding should not be discouraged. Instead, environmental policies should safeguard the exceptionality of BM as nature’s most refined gift for infant development.

We encourage biomonitoring for environmental contaminants, and measures to reduce exposure should be put in place according to local needs/exposures as these vary in different geographical areas depending on human activities. In real life, people are exposed to complex mixtures of EDCs with potentially additive, synergistic, or antagonistic interactions. These mixture effects can disrupt multiple endocrine axes simultaneously, even when individual chemicals are present at low concentrations below regulatory thresholds [33,63]. Moreover, animal studies have shown transgenerational effects of EDC mixture exposure via epigenetic reprogramming [60].

Public health strategies should focus on protecting mothers from environmental exposure to contaminants rather than discouraging breastfeeding. Since many EDCs are persistent with long biological half-lives, achieving a complete elimination from the human body is not feasible, but reducing exposure is possible. Increased public awareness and implementing evidence-based regulatory frameworks that account for mixture effects are essential to reduce exposure and safeguard health.

Summarizing, BM remains the irreplaceable gold standard for infant nutrition, providing unmatched nutritional, immunological, and psychological benefits. Nevertheless, its role as a potential vector of environmental contaminants highlights the pressing need for protective strategies. Future priorities should include:

(1) harmonized biomonitoring of EDCs and micro/nanoplastics in BM;
(2) large-scale, longitudinal cohort studies to clarify exposure–outcome relationships;
(3) public health policies that reduce maternal pollutant exposure without undermining confidence in breastfeeding;
(4) improved regulatory frameworks that address chemical mixture effects and long-term risks.

## Data Availability

When writing the manuscript, the authors did not have access to any special sets of data. As such, the authors cannot provide any special access to datasets that readers might request.
